# Employments for Women

**Published:** 1891-07-18

**Authors:** 


### Employments for Women.

### I.—LADY DRESSMAKERS.

The young lady of the present day is nothing if not enterprising. Not content with trying her hand at what has until lately been considered men's work, she turns her attention to one after another of those employments which have hitherto been the special province of the "young person." And a very good thing it is too; for women of the upper classes are no more all made of one pattern than are women of the lower; and to condemn the "young lady" who must earn her living to but one means of so doing—governessing—is to prevent her—shall we say, in nine cases out of ten?—from making the best of herself, and using the powers which God has given her. By all means, then, let us welcome lady dressmakers, as well as lady nurses and lady doctors. There is plenty of room for them, and plenty of respect too, if they take up their profession in a business-like and sensible way. If the term "lady dressmaker" provokes a smile from some, that is because certain society women have taken up the work more as a pastime than as a serious occupation, and by their unbusiness-like ways, not to speak of their airs and graces, have made themselves the terror or the laughing-stock of their customers. The first thing a lady dressmaker must do is—we were going to say, to forget that she is a lady. Nay, but to forget to insist upon the fact. The woman who tries to show who and what she is by her supercilious and nonchalant airs, stamps herself as of inferior breeding just as much as does the woman who tells you that "she's a lady, she would have you know!"

And the second thing a lady dressmaker must do is—to learn her business thoroughly. A little hap-hazard knowledge, and a few promises of patronage from private friends and benevolent people, may do to start with, but they will never lay the foundations of a business. Anything worth doing is worth doing well, and *must* be done well, if we are to earn a living by it. Some years ago, it might have been difficult for a girl of gentle birth and breeding to learn the trade without exposing herself to certain unpleasantnesses which the constant companionship of girls of a lower grade might bring with it. Now her path has been made very easy to her. There are at least two workrooms, – that under the management of Miss Younghusband, of *Work and Leisure* fame, and that of the Society of Associated Artistes (97, Wimpole Street), in which special arrangements are made for lady pupils—indeed, they were specially started with a view to giving a "thorough training in dressmaking and millinery to gentlewomen by birth and education, wishing to make professional use of their knowledge," the lessons being given "by ladies to ladies." The latter Society (which appears to be a very practical and sensible one, in spite of its amusingly high-falutin' name) has two sets of workrooms, one for the execution of orders, and one for the training of pupils, both being under the direction of ladies. In the latter, amateur pupils are taken, and given simple or advanced courses, for fees varying from two to nine guineas; professional pupils of course go through the whole course of instruction, paying a fee of ten guineas, and are then expected to serve at least three months in the workroom, to gain experience. After this, they may be taken on as permanent hands in the business, or they may, of course, set up for themselves—though such a course needs much care and circumspection, especially if they undertake to supply materials, and thus burden themselves with the worries and anxieties of "stock-keeping." Or, again, a lady may take up the rôle of a "visiting dressmaker," accepting engagements by the week in private houses, where she will probably work with the ladies of the house, and should, of course, be treated as one of themselves. Miss Younghusband makes this a regular part of her business, sending out lady workers into town and country; and it is said to answer very well; though for our part we should prefer, we fancy, the greater independence which employment in a workroom would give.

But wherever the work is done, dressmaking cannot be represented, we fear, as a light and easy employment, if one is to gain a livelihood by it. The hours are long, the slack and busy seasons almost equally trying, one from pressure of work, the other, too often, from pressure of anxiety; the pay (except where "crack" dressmakers are concerned) is but small. As to the hours, the managing directors of the Wimpole Street Workrooms at first tried shorter hours than those at other establishments, but it was found that less than eleven hours (with one hour off for meals) could not be worked without very heavy loss in the busy season. To a girl who has never been used to drudge, and has flitted from one occupation to another at her own sweet will, the long morning—and again the long afternoon—seems as if it would never end; and when she creeps out at lunch time, after four hours spent, say, in buttonhole-making, her back and eyes ache, all her energies flag, and she feels sometimes as if she had no strength or courage left for a little run in the fresh air. Still, she should certainly brace herself up to take it. Dressmakers, of all people, need all the air and exercise that they can get, if they would keep off that ever-ready foe, dyspepsia; and if they could only arrange to attend evening classes for light gymnastics, we are convinced that this would be of the greatest benefit to them.

With regard to the slender earnings with which dressmakers have, as a rule, to be content, there is no doubt that the large number of women who are willing to work at dressmaking for pocket money only, has a considerable effect on the rate of wages, and makes it very difficult for those who live by dressmaking to charge a proper price for their work. Rumour says, indeed, that many dressmakers compensate themselves for the low prices which they are forced to take, by charging excessive prices for linings, &c., or by ordering, and then keeping back for themselves, two or three yards more material than is really needed for the dress. But such expedients, if really resorted to, are by no means satisfactory; and for the credit of the trade, as well as for the comfort and prosperity of the workers, it is much to be wished that dressmakers could form some bond of union with each other, and try what they could do towards putting their affairs on a more satisfactory basis, both with regard to the long hours and to the rate of payment.

